# Supplementary material for: Role of Bacillus cereus in Improving the Growth and Phytoextractability of Brassica nigra (L.) K. Koch in Chromium Contaminated Soil
Source: Molecules. 2021 Mar 12;26(6):1569. doi: 10.3390/molecules26061569 (PMC7998664; doi:10.3390/molecules26061569)
Supplement: Supplementary file 1 [file molecules-26-01569-s001.pdf]

molecules

Supplementary Material

Role of *Bacillus cereus* in Improving the Growth and Phytoextractability of *Brassica nigra* (L.) K. Koch in Chromium Contaminated Soil

Nosheen Akhtar [1], Noshin Ilyas [1*], Humaira Yasmin [2], R. Z. Sayyed [3] , Zuhair Hasnain [4], Elsayed A. Elsayed [5,6], Hesham A. El Enshasy[7,8*]

[1] Department of Botany, PMAS-Arid Agriculture University, 46300, Rawalpindi, Pakistan
[2] Department of Bio-Sciences, COMSATS University, 45550 Islamabad, Pakistan
[3] Department of Microbiology, PSGVP Mandal's Arts, Science and Commerce College, Shahada 425409 Maharashtra, India
[4] Department of Agronomy, PMAS-Arid Agriculture University, 46300, Rawalpindi, Pakistan
[5] Zoology Department, Faculty of Science, King Saud University, Riyadh 11451, Saudi Arabia; eaelsayed@ksu.edu.sa
[6] Natural & Microbial Products Dept., National Research Centre, Dokki, Cairo, Egypt.
[7] Institute of Bioproduct Development (IBD), Universiti Teknologi Malaysia (UTM), Skudai, Johor Bahru, 81310 Johor, Malaysia; zulaiha@ibd.utm.my
[8] City of Scientific Research and Technology Applications, New Burg Al Arab, 21934 Alexandria. Egypt.
* Correspondence: noshinilyas@yahoo.com (N.I.); henshasy@ibd.utm.my (H.E.E.)

Table S1. Analysis of heavy metal concentration in the soil sample collected from the rhizosphere of *Chenopodium album* grown at the contaminated site.

Elements	Heavy metal concentration
Cr	989ppm
Co	26ppm
Ni	103ppm
Zn	276ppm
Pb	214ppm

Table S2. Morphology of bacterial strains isolated from the soil sample collected from rhizosphere of *Chenopodium album* grown in contaminated area.

Isolate Name	Size	Shape	Pigmen -tation	Texture	Margin	Elevation	Gram staining	Optical properties	Appea- rance
B01	small	helical	off white	smooth	regular	convex	Gram positive	opaque	shiny
B02	medium	s-shape	creamy	rough	regular	flat	Gram positive	opaque	shiny
B03	small	vibriod	creamy	rough	irregular	flat	Gram positive	opaque	shiny
B04	small	helical	white	smooth	irregular	flat	Gram	opaque	shiny

							positive		
B05	large	rod	creamy	rough	irregular	circular	Gram positive	opaque	shiny
B06	medium	s-shape	yellowish	rough	regular	convex	Gram positive	opaque	shiny
B07	small	rod	white	rough	irregular	convex	Gram positive	opaque	dull
B08	medium	helical	white	smooth	irregular	circular	Gram positive	opaque	shiny
B09	medium	helical	off-white	rough	irregular	convex	Gram negative	opaque	shiny
B10	large	rod	creamy	rough	regular	flat	Gram positive	opaque	shiny
B11	medium	rod	off-white	smooth	irregular	flat	Gram positive	opaque	shiny
B12	small	rod	yellowish	rough	irregular	circular	Gram positive	opaque	shiny
B13	medium	s-shape	creamy	smooth	regular	circular	Gram negative	opaque	shiny
B14	large	rod	creamy	smooth	irregular	flat	Gram positive	opaque	shiny
B15	large	vibriod	white	smooth	irregular	convex	Gram negative	opaque	dull

Table S3. Analysis of heavy metal tolerance (chromium) of bacterial stains.

Isolate name	growth at Cr-20mg/L	growth at Cr-30mg/L	growth at Cr-40mg/L
B01	+	-	-
B02	+	-	-
B03	+	+	+
B04	+	-	-
B05	+	+	+
B06	+	-	-
B07	+	+	+
B08	+	-	-
B09	+	-	-
B10	+	+	+
B11	+	-	-
B12	+	+	-
B13	+	-	-

B14	+	+	-
B15	+	-	-

Table S4. Analysis of plant growth-promoting characteristics of bacterial strains.

Isolate name	Siderophore production	ACC deaminase synthesis	Phosphate solubilization	organic acid synthesis
B01	+	+	+	-
B02	+	-	-	-
B03	+	+	+	+
B04	+	-	+	-
B05	+	+	+	+
B06	+	-	-	+
B07	-	-	+	-
B08	+	-	+	+
B09	-	+	+	-
B10	-	+	+	-
B11	-	+	-	-
B12	-	-	-	+
B13	+	-	+	+
B14	-	+	-	+
B15	+	-	+	-

Table S5. Molecular identification of bacterial strain by 16srRNA sequencing.

Isolate	Strain Identification	Accession No.	Base Pair Length
NA05	*Bacillus subtilis*	MW316052	1398 bp
